# Supplementary material for: MicroRNA-122 supports robust innate immunity in hepatocytes by targeting the RTKs/STAT3 signaling pathway
Source: eLife. 2019 Feb 8;8:e41159. doi: 10.7554/eLife.41159 (PMC6389286; doi:10.7554/eLife.41159)
Supplement: Supplementary file 1. [file elife-41159-supp1.docx]

Supplementary File 1. The 330 candidate STAT3 regulators.

| **Classification** | **Family** | **Gene** |
| --- | --- | --- |
| Tyrosine kinase | JAK Family | JAK1, JAK2, JAK3, TYK2 |
|  | Receptor  Tyrosine Kinase (RTK) | AATK, ALK, AXL, CSF1R, DDR1, DDR2, EGFR, EPHA1, EPHA10, EPHA2, EPHA3, EPHA4, EPHA5, EPHA6, EPHA7, EPHA8, EPHB1, EPHB2, EPHB3, EPHB4, EPHB6, ERBB2, ERBB3, ERBB4, FGFR1, FGFR2, FGFR3, FGFR4, FLT1, FLT3, FLT4, IGF1R, INSR, INSRR, KDR, KIT, LMTK2, LMTK3, LTK, MERTK, MET, MST1R, MUSK, NTRK1, NTRK2, NTRK3, PDGFRA, PDGFRB, PTK7, RET, ROR1, ROR2, ROS1, RYK, STYK1, TEK, TIE1, TYRO3 |
|  | N-RTK | ABL1, ABL2, BLK, BMX, BTK, CSK, DSTYK, FER, FES, FYN, HCK, LCK, LYN, MATK, PKM2, PTK2, PTK2B, PTK6, SRC, SYK, TEC, TNK1, TNK2, TXK, YES1 |
| RTK Ligand | RTK Ligand | ANGPT2, BDNF, CSF1, EFNA1, EFNA2, EFNA3, EFNA4, EFNA5, EFNB1, EFNB2, EFNB3, EGF, EGFL6, EGFL7, EGFL8, EGFLAM, FGF1, FGF10, FGF11, FGF12, FGF13, FGF14, FGF16, FGF17, FGF18, FGF19, FGF2, FGF20, FGF21, FGF22, FGF23, FGF3, FGF4, FGF5, FGF6, FGF7, FGF8, FGF9, FLT3LG, GAS6, HGF, IGF1, IGF2 , IGFL1, IGFL2, KGFLP1 /// KGFLP2, KITLG, NAMPT, PDGFA, PDGFB, PDGFC, PDGFD, VEGFA, VEGFB, VEGFC |
|  | Potential RTK Ligand | ANGPT1, ANGPT4, ANGPTL1, ANGPTL2, ANGPTL3, ANGPTL4, ANGPTL6, ANGPTL7, PDGFRL |
| Cytokine & Hormone | IL6 Family | CLCF1, CNTF, CSF3, CTF1, IL11, IL6, LEP, LIF, OSM |
|  | IL10 Family | IL10, IL19, IL20, IL22, IL24, IL26 |
|  | IL12 Family | IL12A, IL12B, IL23A, IL27 |
|  | CbCRF | CSF2, IL3, IL5, IL15, IL17A, IL17B, IL17C, IL17D, IL17F, IL2, IL25, IL4, IL7, IL9, TSLP, IL13 |
|  | Interferon | IFNA1, IFNA10, IFNA13, IFNA14, IFNA16, IFNA17, IFNA2, IFNA21, IFNA4, IFNA5, IFNA6, IFNA7, IFNA8, IFNB1, IFNE, IFNG, IFNK, IFNW1, IL28A, IL28B, IL29 |
|  | Other Interleukin | IL16, IL18, IL1A, IL1B, IL1F10, IL1F5, IL1F6, IL1F7, IL1F8, IL1F9, IL1RN, IL21, IL32, IL33, IL34, IL8 |
|  | GPCR Ligand | CCL14 /// CCL15, CCL2, CCL5, CXCL12, F2 |
|  | Homodimeric Hormone (HH) | EPO, GH1, GH2, PRL, THPO, TPO |
| Cytokine & Hormone Receptor | IL6 Family Receptor | CNTFR, CSF3R, IL11RA, IL31RA, IL6R, IL6ST, LEPR, LIFR, OSMR |
|  | IL10 Family Receptor | IL10RA, IL10RB, IL20RA, IL20RB, IL22RA1, IL22RA2 |
|  | IL12 Family Receptor | IL12RB1, IL12RB2, IL23R, IL27RA |
|  | CbCRF Family Receptor | IL3RA, IL5RAIL13RA1, IL13RA2, IL15RA, IL17RA, IL17RB, IL17RC, IL17RD, IL17RE, IL2RA, IL2RB, IL2RG, IL4R, IL7R, IL9R |
|  | Interferon Receptor | IFNAR1, IFNAR2, IFNGR1, IFNGR2, IL28RA |
|  | Other Cytokine Receptor | IL18R1, IL1R1, IL1R2, IL1RL1, IL1RL2, IL21R, IL8RA, IL8RB |
|  | GPCR | ADRA1A, ADRA1B, ADRA1D, AGTR1, AGTR2, BDKRB1, BDKRB2, CCR1, CCR2, CCR5, CXCR4, F2R, HTR2A, PTAFR |
|  | HHR | EPOR, GHR, MPL, PRLR |
| Other Regulators | Other Known Regulator | CHUK, CNKSR1, COPB2, CSNK2A2, DGKB, DYRK4, GFRA2, HUNK, ILK, MAP3K13, MAP3K2, MAP3K3, MAP3K7, MAPK4, NEK8, NPR1, PFKFB1, PICK1, RAPGEF3, TEX14, TTBK2, UCK1 |
|  | Cytokine-receptor interaction | IL18BP, IL18RAP, IL1RAP, IL1RAPL1, IL1RAPL2 |
|  | RTK-related protein | CSF2RA, CSF2RB, IGF2R |
