# Supplementary material for: MicroRNA-122 supports robust innate immunity in hepatocytes by targeting the RTKs/STAT3 signaling pathway
Source: eLife. 2019 Feb 8;8:e41159. doi: 10.7554/eLife.41159 (PMC6389286; doi:10.7554/eLife.41159)
Supplement: Supplementary file 2. [file elife-41159-supp2.docx]

Supplementary File 2. The expression of 25 candidate STAT3 activators in microarray data.

| **ProbeSetID** | **PublicID** | **HepG2_miR-122_M** | **HepG2_NC_M** | **Ratio** | **HepG2_miR-122_ON** | **HepG2_miR-122_OFF** | **Ratio** |
| --- | --- | --- | --- | --- | --- | --- | --- |
| 212657_s_at | IL1RN | 44.67135 | 112.4622 | 0.3972 | 35.32422 | 61.51567 | 0.5742 |
| 211506_s_at | IL8 | 25.9222 | 38.30464 | 0.6767 | 16.34402 | 25.34317 | 0.6449 |
| 202859_x_at | IL8 | 117.8331 | 172.4226 | 0.6834 | 62.61566 | 118.9641 | 0.5263 |
| 202948_at | IL1R1 | 218.3936 | 380.7611 | 0.5736 | 366.7867 | 631.0038 | 0.5813 |
| 215561_s_at | IL1R1 | 14.02876 | 20.20374 | 0.6944 | 12.52875 | 22.07771 | 0.5675 |
| 209962_at | EPOR | 12.84181 | 17.34032 | 0.7406 | 8.229953 | 10.88412 | 0.7561 |
| 207257_at | EPO | 50.95065 | 85.24219 | 0.5977 | 30.643 | 47.6946 | 0.6425 |
| 217254_s_at | EPO | 39.74287 | 49.85342 | 0.7972 | 20.8301 | 36.11709 | 0.5767 |
| 227771_at | LIFR | 12.53784 | 20.55051 | 0.6101 | 22.86204 | 30.04452 | 0.7609 |
| 205729_at | OSMR | 33.64029 | 47.07159 | 0.7147 | 49.55137 | 63.23963 | 0.7835 |
| 244261_at | IL28RA | 101.1069 | 132.6588 | 0.7622 | 86.65221 | 109.1018 | 0.7942 |
| 227677_at | JAK3 | 16.7748 | 21.80758 | 0.7692 | 21.32544 | 32.70694 | 0.652 |
| 226893_at | ABL2 | 89.46267 | 148.305 | 0.6032 | 80.37595 | 104.9363 | 0.7659 |
| 231907_at | ABL2 | 86.35794 | 118.3571 | 0.7296 | 78.50414 | 113.4178 | 0.6922 |
| 229017_s_at | DSTYK | 22.5598 | 53.71951 | 0.42 | 26.35416 | 40.2668 | 0.6545 |
| 201251_at | PKM2 | 578.8014 | 968.1644 | 0.5978 | 392.7694 | 594.8014 | 0.6603 |
| 227131_at | MAP3K3 | 132.1935 | 247.5444 | 0.534 | 110.7024 | 162.6386 | 0.6807 |
| 211535_s_at | FGFR1 | 416.3761 | 762.4271 | 0.5461 | 237.5487 | 403.8105 | 0.5883 |
| 226705_at | FGFR1 | 251.9116 | 433.1011 | 0.5816 | 157.1462 | 245.0024 | 0.6414 |
| 222164_at | FGFR1 | 55.61632 | 95.17491 | 0.5844 | 40.69948 | 60.53429 | 0.6723 |
| 203628_at | IGF1R | 218.0352 | 280.5113 | 0.7773 | 120.6887 | 160.3014 | 0.7529 |
| 211913_s_at | MERTK | 45.89385 | 106.4559 | 0.4311 | 45.46998 | 61.78888 | 0.7359 |
| 233079_at | MERTK | 43.48627 | 61.88791 | 0.7027 | 41.0715 | 52.20199 | 0.7868 |
| 202023_at | EFNA1 | 858.3703 | 1291.801 | 0.6645 | 643.0514 | 840.3472 | 0.7652 |
| 227271_at | FGF11 | 59.18624 | 86.19915 | 0.6866 | 43.17839 | 61.38929 | 0.7034 |
| 210881_s_at | IGF2 | 86.47013 | 165.461 | 0.5226 | 70.38568 | 104.5096 | 0.6735 |
| 202410_x_at | IGF2 | 155.0449 | 276.6834 | 0.5604 | 104.5314 | 175.7384 | 0.5948 |
| 202409_at | IGF2 | 1681.578 | 2517.545 | 0.6679 | 1091.516 | 1811.386 | 0.6026 |
| 217738_at | NAMPT | 54.53153 | 79.9601 | 0.682 | 90.03673 | 113.3038 | 0.7946 |
| 206924_at | IL11 | 63.81179 | 105.2063 | 0.6065 | 62.53775 | 98.30137 | 0.6362 |
| 205723_at | CNTFR | 24.44045 | 34.07955 | 0.7172 | 27.10279 | 35.94448 | 0.754 |
| 203638_s_at | FGFR2 | 11.41559 | 16.57408 | 0.6888 | 13.96427 | 19.62115 | 0.7117 |
| 206462_s_at | NTRK3 | 5.425767 | 7.734156 | 0.7015 | 5.856435 | 7.743003 | 0.7564 |
| 231382_at | FGF18 | 7.728011 | 10.60155 | 0.729 | 6.393689 | 9.562734 | 0.6686 |

Five genes in gray background were undetected (FGFR2, NTRK3 and FGF18) or unchanged (IL11 and CNTFR) in qRT-PCR assays.
