# Supplementary material for: MicroRNA-122 supports robust innate immunity in hepatocytes by targeting the RTKs/STAT3 signaling pathway
Source: eLife. 2019 Feb 8;8:e41159. doi: 10.7554/eLife.41159 (PMC6389286; doi:10.7554/eLife.41159)
Supplement: Supplementary file 3. — The candidate miR-122 targets and binding sites were predicted by starbase (http://starbase.sysu.edu.cn/). The targets shown are 47 genes from among the 330 candidate STAT3 regulators. [file elife-41159-supp3.docx]

Supplementary File 3. Candidate STAT3 activators that predicted to be miR-122 targets in published CLIP-seq data.

| geneName | chromosome | narrowStart | narrowEnd | strand | clipExpNum | RBP |
| --- | --- | --- | --- | --- | --- | --- |
| ABL1 | chr9 | 133761535 | 133761540 | + | 7 | AGO1-4,AGO2 |
| ABL1 | chr9 | 133762063 | 133762068 | + | 7 | AGO1-4,AGO2 |
| ABL2 | chr1 | 179072263 | 179072268 | - | 3 | AGO1-4,AGO2 |
| ABL2 | chr1 | 179069813 | 179069818 | - | 2 | AGO1-4,AGO2 |
| ABL2 | chr1 | 179073152 | 179073157 | - | 2 | AGO1-4 |
| ABL2 | chr1 | 179070560 | 179070565 | - | 1 | AGO1-4 |
| ANGPTL4 | chr19 | 8439021 | 8439026 | + | 1 | AGO1-4 |
| CHUK | chr10 | 101948534 | 101948539 | - | 2 | AGO2 |
| CSF1 | chr1 | 110466276 | 110466295 | + | 2 | AGO1-4,AGO2 |
| CXCL12 | chr10 | 44866431 | 44866437 | - | 3 | AGO1-4 |
| CXCL12 | chr10 | 44868490 | 44868495 | - | 3 | AGO1-4 |
| CXCL12 | chr10 | 44876231 | 44876253 | - | 2 | AGO1-4 |
| DDR2 | chr1 | 162725020 | 162725048 | + | 1 | AGO1-4 |
| DDR2 | chr1 | 162750111 | 162750116 | + | 1 | AGO1-4 |
| DDR2 | chr1 | 162750184 | 162750189 | + | 1 | AGO1-4 |
| DSTYK | chr1 | 205116587 | 205116593 | - | 7 | AGO1-4,AGO2 |
| DSTYK | chr1 | 205138857 | 205138885 | - | 3 | AGO1,AGO2 |
| EFNA1 | chr1 | 155107107 | 155107112 | + | 10 | AGO1-4,AGO2 |
| EFNA1 | chr1 | 155106597 | 155106602 | + | 3 | AGO1-4 |
| EFNA1 | chr1 | 155106647 | 155106652 | + | 3 | AGO1-4 |
| EFNA3 | chr1 | 155059517 | 155059522 | + | 5 | AGO1-4,AGO2 |
| EFNB2 | chr13 | 107144837 | 107144842 | - | 1 | AGO1-4 |
| EPHB2 | chr1 | 23240858 | 23240863 | + | 1 | AGO1-4 |
| ERBB3 | chr12 | 56496113 | 56496118 | + | 5 | AGO1-4,AGO2 |
| ERBB3 | chr12 | 56497062 | 56497067 | + | 3 | AGO1-4 |
| ERBB4 | chr2 | 212241811 | 212241816 | - | 1 | AGO2 |
| FGF18 | chr5 | 170883851 | 170883857 | + | 1 | AGO2 |
| FGF5 | chr4 | 81208029 | 81208036 | + | 2 | AGO1-4 |
| FGFR1 | chr8 | 38269188 | 38269193 | - | 4 | AGO1-4,AGO2 |
| FGFR3 | chr4 | 1809054 | 1809059 | + | 2 | AGO1-4 |
| IFNGR2 | chr21 | 34805123 | 34805151 | + | 5 | AGO1,AGO1-4,AGO2 |
| IFNGR2 | chr21 | 34793920 | 34793948 | + | 1 | AGO1-4 |
| IGF1R | chr15 | 99502018 | 99502023 | + | 14 | AGO1,AGO1-4,AGO2 |
| IGF1R | chr15 | 99501368 | 99501374 | + | 8 | AGO1-4,AGO2 |
| IGF1R | chr15 | 99505442 | 99505447 | + | 3 | AGO1-4,AGO2 |
| IGF1R | chr15 | 99505630 | 99505635 | + | 3 | AGO1-4,AGO2 |
| IGF2R | chr6 | 160526279 | 160526284 | + | 8 | AGO1-4,AGO2 |
| IL18 | chr11 | 112014096 | 112014101 | - | 1 | AGO1-4 |
| IL18BP | chr11 | 71713220 | 71713225 | + | 2 | AGO1-4,AGO2 |
| IL18BP | chr11 | 71713299 | 71713304 | + | 2 | AGO1-4,AGO2 |
| IL18BP | chr11 | 71714065 | 71714070 | + | 1 | AGO1-4 |
| IL1R1 | chr2 | 102794423 | 102794428 | + | 3 | AGO1-4,AGO2 |
| IL1R1 | chr2 | 102794523 | 102794528 | + | 3 | AGO1-4,AGO2 |
| IL1R1 | chr2 | 102795048 | 102795053 | + | 1 | AGO1-4 |
| IL1RL1 | chr2 | 102960090 | 102960095 | + | 1 | AGO1-4 |
| IL1RN | chr2 | 113891211 | 113891216 | + | 1 | AGO1-4 |
| IL6ST | chr5 | 55238505 | 55238512 | - | 1 | AGO1-4 |
| IL7R | chr5 | 35876260 | 35876282 | + | 1 | AGO1-4 |
| JAK1 | chr1 | 65299985 | 65299990 | - | 4 | AGO1-4,AGO2 |
| KITLG | chr12 | 88887852 | 88887857 | - | 3 | AGO1-4,AGO2 |
| LIF | chr22 | 30637827 | 30637832 | - | 1 | AGO1-4 |
| LIF | chr22 | 30639489 | 30639494 | - | 1 | AGO1-4 |
| MAP3K2 | chr2 | 128062281 | 128062287 | - | 2 | AGO1-4,AGO2 |
| MAP3K3 | chr17 | 61773179 | 61773184 | + | 6 | AGO1-4,AGO2 |
| MAP3K3 | chr17 | 61772140 | 61772145 | + | 1 | AGO1-4 |
| MAPK4 | chr18 | 48257044 | 48257049 | + | 7 | AGO1,AGO2 |
| MAPK4 | chr18 | 48257737 | 48257743 | + | 1 | AGO2 |
| MET | chr7 | 116436405 | 116436411 | + | 4 | AGO1-4,AGO2 |
| MST1R | chr3 | 49924585 | 49924590 | - | 1 | AGO2 |
| NTRK2 | chr9 | 87366957 | 87366985 | + | 2 | AGO1-4,AGO2 |
| OSMR | chr5 | 38933716 | 38933722 | + | 2 | AGO1-4,AGO2 |
| PDGFA | chr7 | 537110 | 537115 | - | 4 | AGO1-4,AGO2 |
| PDGFB | chr22 | 39620590 | 39620597 | - | 2 | AGO1-4 |
| PDGFB | chr22 | 39620450 | 39620455 | - | 1 | AGO1-4 |
| PRLR | chr5 | 35065200 | 35065206 | - | 8 | AGO1-4,AGO2 |
| PRLR | chr5 | 35064742 | 35064748 | - | 4 | AGO1-4,AGO2 |
| PRLR | chr5 | 35059984 | 35059989 | - | 1 | AGO1-4 |
| RET | chr10 | 43624973 | 43624979 | + | 3 | AGO1-4 |
| ROR1 | chr1 | 64644736 | 64644764 | + | 2 | AGO1,AGO3 |
| ROR2 | chr9 | 94485606 | 94485611 | - | 1 | AGO2 |
| SRC | chr20 | 36032035 | 36032040 | + | 1 | AGO2 |
| TEX14 | chr17 | 56650552 | 56650574 | - | 1 | AGO1-4 |
| VEGFB | chr11 | 64004969 | 64004991 | + | 6 | AGO1-4,AGO2 |

The candidate miR-122 targets and the binding sites were predicted by starbase (<http://starbase.sysu.edu.cn/>). The targets shown are 47 genes that fall in the 330 candidate STAT3 regulators.
