# Supplementary material for: MicroRNA-122 supports robust innate immunity in hepatocytes by targeting the RTKs/STAT3 signaling pathway
Source: eLife. 2019 Feb 8;8:e41159. doi: 10.7554/eLife.41159 (PMC6389286; doi:10.7554/eLife.41159)
Supplement: Supplementary file 4. [file elife-41159-supp4.docx]

Supplementary File 4. Oligonucleotides.

| **Oligo Name** | **Sequence (5′ to 3′)** | **Experiments** |
| --- | --- | --- |
| RT-miR-122-5p | CTCAACTGGTGTCGTGGAGTCGGCAATTCAGTTGAGCAAACACC | RT |
| FP-miR-122-5p | TCGCCTGGAGTGTGACAATGG | qPCR |
| UNIV-RP | CTCAACTGGTGTCGTGGAGTC | qPCR |
| U6-F | CGCTTCGGCAGCACATATAC | qPCR |
| U6-R | TTCACGAATTTGCGTGTCAT | qPCR |
| GAPDH-F1 | CCATGGGGAAGGTGAAGGTC | qPCR |
| GAPDH-R1 | GAAGGGGTCATTGATGGCAAC | qPCR |
| IL28-F | CTTTAAGAGGGCCAAAGATGC | qPCR |
| IL28-R | CCAGCTCAGCCTCCAAAG | qPCR |
| IL29-F | ATTGGGACCTGAGGCTTCTC | qPCR |
| IL29-R | TGAAGGGGCTGGTCTAGGA | qPCR |
| IFNB1-F | CCTGAAGGCCAAGGAGTACA | qPCR |
| IFNB1-R | AAGCAATTGTCCAGTCCCAG | qPCR |
| RIGI-F | TGCAAGCCTTCCAGGATTAT | qPCR |
| RIGI-R | ATTGGGCCCTTGTTGTTTTT | qPCR |
| MDA5-F | TTCAACCACAGTTCAGCCAA | qPCR |
| MDA5-R | TGACACTTCCTTCTGCCAAA | qPCR |
| CXCL9-F | CCTTAAACAATTTGCCCCAA | qPCR |
| CXCL9-R | TTTGGCTGACCTGTTTCTCC | qPCR |
| CXCL10-F | GAGCCTACAGCAGAGGAACC | qPCR |
| CXCL10-R | GCTGATGCAGGTACAGCGT | qPCR |
| HBV-pgF | TGAGCATTGTTCACCTCACC | qPCR |
| HBV-pgR | CACACTCCGAAAGACACCAA | qPCR |
| HCV-F | GGCGACACTCCGCCATGAATC | qPCR |
| HCV-R | TCAGGCAGTACCACAAGGC | qPCR |
| HCV-RT-1 | CCTCCCAAAATTCAAGATGGTC | RT |
| SOCS1-F | AGAGCTTCGACTGCCTCTTC | qPCR |
| SOCS1-R | AGGGGAAGGAGCTCAGGTAG | qPCR |
| SOCS2-F | ATAAGCGGACAGGTCCAGAA | qPCR |
| SOCS2-R | ATGGCACCGGTACATTTGTT | qPCR |
| SOCS3-F | GCCACCTACTGAACCCTCCT | qPCR |
| SOCS3-R | ACGGTCTTCCGACAGAGATG | qPCR |
| SOCS4-F | GATAAATACGCAGCCGAAGC | qPCR |
| SOCS4-R | GGAGCCCAGTAATGTCAGGA | qPCR |
| SOCS5-F | ACCCAGAGTTCATTGGATGC | qPCR |
| SOCS5-R | CCCACAGTATCCTGCAACCT | qPCR |
| STAT3-F | GAGCTGGCTGACTGGAAGAG | qPCR |
| STAT3-R | TGTTGACGGGTCTGAAGTTG | qPCR |
| IRF1-F | GACCCTGGCTAGAGATGCAG | qPCR |
| IRF1-R | AGGCATCCTTGTTGATGTCC | qPCR |
| IRF3-F | GATGCACAGCAGGAGGATTT | qPCR |
| IRF3-R | TAAACGCAACCCTTCTTTGC | qPCR |
| IRF7-F | CCCCATCTTCGACTTCAGAG | qPCR |
| IRF7-R | TGCTGCTATCCAGGGAAGAC | qPCR |
| p65(RELA)-F | CTCCTGTGCGTGTCTCCAT | qPCR |
| p65(RELA)-R | TTTCTCCTCAATCCGGTGAC | qPCR |
| p50(NFKB1)-F | ATGTATGTGAAGGCCCATCC | qPCR |
| p50(NFKB1)-R | ATAACCTTTGCTGGTCCCAC | qPCR |
| IL1RN-F | TTGCAAGGACCAAATGTCAA | qPCR |
| IL1RN-R | TCTCGCTCAGGTCAGTGATG | qPCR |
| MERTK-F | GGGTCCAGAACCATGAGATG | qPCR |
| MERTK-R | TCTAAGGGATCGGTTCTCCA | qPCR |
| EPO-F | TCACTGTCCCAGACACCAAA | qPCR |
| EPO-R | GGAAGAGTTGACCAACAGGG | qPCR |
| LIFR-F | TCCACACCGCTCAAATGTTA | qPCR |
| LIFR-R | TTCCAAGGGCATATCTGAGG | qPCR |
| FGFR1-F | CACATCCAGTGGCTAAAGCA | qPCR |
| FGFR1-R | ATAGAGTTACCCGCCAAGCA | qPCR |
| JAK3-F | ATTCAACAGTGCATGGCCTA | qPCR |
| JAK3-R | CATAGAGCTGGGCACCATTC | qPCR |
| PKM2-F | CTATCCTCTGGAGGCTGTGC | qPCR |
| PKM2-R | CACTGCAGCACTTGAAGGAG | qPCR |
| IGF2-F | AGCTGGTGGACACCCTCCAGTTC | qPCR |
| IGF2-R | TCGACACGTCCCTCTCGGACTTG | qPCR |
| IL8-F | CAAGAGCCAGGAAGAAACCA | qPCR |
| IL8-R | AGCACTCCTTGGCAAAACTG | qPCR |
| DSTYK-F | GACATCAAGTGCTCCCACAA | qPCR |
| DSTYK-R | TAGGTGGGAAGGAAATGCAG | qPCR |
| ABL2-F | GAGCTCCAAGGAGAACTTGC | qPCR |
| ABL2-R | TGCCCATTCTTAGAGCGAAC | qPCR |
| MAP3K3-F | GAAGCAGGCACCTCTCTGTC | qPCR |
| MAP3K3-R | GAACTCCCCCTCACTGTTGA | qPCR |
| FGF11-F | CAGAAGCAGCTCCTCATCCT | qPCR |
| FGF11-R | GCAGAACAGTTTGGTGACGA | qPCR |
| EFNA1-F | GCTATGGAGTTCCTCTGGGC | qPCR |
| EFNA1-R | TCAGCTGCACATGTATGGTGT | qPCR |
| IL1R1-F | AGCTGGACCCCTTGGTAAAA | qPCR |
| IL1R1-R | TTCTTCACGTTCCTTGCATTT | qPCR |
| NAMPT-F | GCAGAAGCCGAGTTCAACAT | qPCR |
| NAMPT-R | TCTGTCTTCTTTTCACGGCA | qPCR |
| IL28RA-F | GTGGAGTCCGAATACCTGGA | qPCR |
| IL28RA-R | CTTCCAGAATGCCACCTCAT | qPCR |
| IGF1R-F | AGCCGATGTGTGAGAAGACC | qPCR |
| IGF1R-R | GTGGCAGCACTCATTGTTCT | qPCR |
| EPOR-F | CTCATCCTCGTGGTCATCCT | qPCR |
| EPOR-R | CTTCAAACTCGCTCTCTGGG | qPCR |
| OSMR-F | CGCGTCAGAGTTTGCACTTA | qPCR |
| OSMR-R | CCACTTCACAGTGGTGCTGT | qPCR |
| IRF1-ChF1 | TTCGCCGCTAGCTCTACAAC | ChIP-PCR |
| IRF1-ChR1 | TTGCCTCGACTAAGGAGTGG | ChIP-PCR |
| IRF1-ChF2 | AGGGGGCTGAGTTCTCTTTC | ChIP-PCR |
| IRF1-ChR2 | GGAAAATCACTAGGGGGTCA | ChIP-PCR |
| IRF1-ChF3 | GTGGGGAGGAATAGGTAGGG | ChIP-PCR |
| IRF1-ChR3 | CCAACTTCCACATTCCTGCT | ChIP-PCR |
| IRF1-ChF4 | ACTGGCCGGTAACACTGGAC | ChIP-PCR |
| IRF1-ChR4 | CCATCCAGTGGGGGTCAG | ChIP-PCR |
| IRF1-ChF5 | AGGTTGAGTCACCAGGGTTG | ChIP-PCR |
| IRF1-ChR5 | CAGCTAGCCAGTGGAAGGAG | ChIP-PCR |
| IRF1-ChF6 | TGCATTTCCCGAACTAGCAG | ChIP-PCR |
| IRF1-ChR6 | GGCTTGACTGGTGGGAAAG | ChIP-PCR |
| IRF1-ChF7 | CAGCCTGAAGGACTCCCTTT | ChIP-PCR |
| IRF1-ChR7 | GAGGCCAAAAGGCAATATGA | ChIP-PCR |
| IRF1-P1-F | TTTGGTACCAGCCGGCTGGGACAAGGCGGAG | Reporter construct |
| IRF1-P1-R | TTTAAGCTTCTCCGGGTGGCCTCGGTTCGGC | Reporter construct |
| IRF1-P2-F | TTTGGTACCGGGGAGGAGAGTGCTGATCCCA | Reporter construct |
| IRF1-P2-R | TTTAAGCTTTGGAAAATCACTAGGGGGTCAT | Reporter construct |
| IRF1-P3-F | TTTGGTACCACCCGGCTGCAAACCCATGGCC | Reporter construct |
| IRF1-P3-R | TTTAAGCTTGCCCCGGCCAGAACTTGGTGG | Reporter construct |
| IRF1-P4-F | TTTGGTACCTCAGGTGGGGCTTTTCCGGGGC | Reporter construct |
| IRF1-P4-R | TTTAAGCTTGGGGCCGGGTAGGGGAGGGCTT | Reporter construct |
| IRF1-P5-F | TTTGGTACCGGGCCACAGGAGAAAGAAGGGA | Reporter construct |
| IRF1-P5-R | TTTAAGCTTTCAGACTAAATGGAGCTCCCTC | Reporter construct |
| IRF1-P6-F | TTTGGTACCTTTCAGCCTGAAGGACTCCCTT | Reporter construct |
| IRF1-P6-R | TTTAAGCTTACCCCAAGTAGGATAAACTCAG | Reporter construct |
| IRF1-P7-F | TTTGGTACCCTTGGTCCCTGTAGCAAGTCC | Reporter construct |
| IRF1-P7-R | TTTCTCGAGTGCTGCAGGAGCGATTCGGC | Reporter construct |
| IRF1-P8-R | TTTAAGCTTAGGCCTGCTTTTTGGTGAACC | Reporter construct |
| IRF1-P1-MF | TACAACAGCCTGATACTACTACTATGACGGCACGCAGCCGGCC | Reporter construct |
| IRF1-P1-MR | CTGCGTGCCGTCATAGTAGTAGTATCAGGCTGTTGTAGAGCTA | Reporter construct |
| IRF1-P4-MF | CCAGCGTGGTTCGGACTACTACTACTCCTCGATTTCCCTGTTG | Reporter construct |
| IRF1-P4-MR | GGAAATCGAGGAGTAGTAGTAGTCCGAACCACGCTGGGAGCGC | Reporter construct |
| P3-IRF1-F | TTTGGATCCGCCACCATGCCCATCACTCGGATGCGCATG | Protein expression |
| P3-IRF1-R | TTTCTCGAGCTACGGTGCACAGGGAATGGCCTG | Protein expression |
| HA-IRF1-R | TTTCTCGAGCGGTGCACAGGGAATGGCCTG | Protein expression |
| HA-IRF3-F | TTTGTCGACAATGGGAACCCCAAAGCCACGGAT | Protein expression |
| HA-IRF3-R | TTTGCGGCCGCTTATTGGTTGAGGTGGTGGGGAAC | Protein expression |
| HA-IRF7-F | TTTGTCGACAATGGCCTTGGCTCCTGAGAGG | Protein expression |
| HA-IRF7-R | TTTGCGGCCGCTTCTAGGCGGGCTGCTCCAGCT | Protein expression |
| HA-p65-F | TTTCTCGAGAATGGACGAACTGTTCCCCCTCAT | Protein expression |
| HA-p65-R | TTTGCGGCCGCTTAGGAGCTGATCTGACTCAGCAG | Protein expression |
| HA-p50-F | TTTGTCGACAATGGCAGAAGATGATCCATATTTGG | Protein expression |
| HA-p50-R | TTTGCGGCCGCTTAAGGGTCCTTTTTAGATTCAGTGTC | Protein expression |
| HA-ATF2-F | TTTGTCGACTATGAGTGATGACAAACCCTTTC | Protein expression |
| HA-ATF2-R | TTTGCGGCCGCTCAACTTCCTGAGGGCTGTGAC | Protein expression |
| HA-JUN-F | TTTGTCGACTATGACTGCAAAGATGGAAACGA | Protein expression |
| HA-JUN-R | TTTGCGGCCGCTCAAAATGTTTGCAACTGCTGCG | Protein expression |
| STAT3-CDS-F | TTTAAGCTTCGCCACCATGGCCCAATGGAATCAGCTACAG | Protein expression |
| STAT3-CDS-R | TTTCTCGAGTCACATGGGGGAGGTAGCGCACTCC | Protein expression |
| HA-JAK1-F | TTTGGTACCGCCACCATGCAGTATCTAAATATAAAAGAGG | Protein expression |
| HA-JAK1-R | TTTCTCGAGTTTTAAAAGTGCTTCAAATCCTTC | Protein expression |
| HA-JAK3-F | TTTGGTACCGCCACCATGGCACCTCCAAGTGAAGAGAC | Protein expression |
| HA-JAK3-R | TTTCTCGAGTGAAAAGGACAGGGAGTGGTGTTT | Protein expression |
| FL-EGFR-F | TTTGTCGACAATGCGACCCTCCGGGACGGCC | Protein expression |
| FL-EGFR-R | TTTGCGGCCGCGTCATGCTCCAATAAATTCACTGC | Protein expression |
| FL-FGFR1-F | TTTGTCGACTATGGAGGCAAGGGTCAGTTTGA | Protein expression |
| FL-FGFR1-R | TTTGCGGCCGCTCAGCGGCGTTTGAGTCCGCCA | Protein expression |
| FL-IGF1R-F | TTTGTCGACTATGAAGTCTGGCTCCGGAGGAGG | Protein expression |
| FL-IGF1R-R | TTTGCGGCCGCTCAGCAGGTCGAAGACTGGGGCAG | Protein expression |
| FL-MERTK-F | TTTGTCGACTATGGGGCCGGCCCCGCTGCCGCTG | Protein expression |
| FL-MERTK-R | TTTGCGGCCGCTCACATCAGGACTTCTGAGCCTTCTGA | Protein expression |
| FL-ABL2-F | TTTGTCGACTATGGTCCTTGGGACAGTTCTCCT | Protein expression |
| FL-ABL2-R | TTTGCGGCCGCCTACCTCTGCACCACATCACTGAT | Protein expression |
| FL-PKM2-F | TTTGTCGACAATGTCGAAGCCCCATAGTGAAG | Protein expression |
| FL-PKM2-R | TTTGCGGCCGCTCACGGCACAGGAACAACACGC | Protein expression |
| HA -IL1RN-F | TTTAAGCTTCGCCACCATGGAAATCTGCAGAGGCCTCCGC | Protein expression |
| HA-IL1RN-R | TTTCTCGAGCTCGTCCTCCTGGAAGTAGAAT | Protein expression |
| HA-EPO-F | TTTGGATCCGCCACCATGGGGGTGCACGAATGTCCTG | Protein expression |
| HA-EPO-R | TTTCTCGAGTCTGTCCCCTGTCCTGCAGG | Protein expression |
| HA-FGF11-F | TTTGGATCCGCCACCATGGCGGCGCTGGCCAGTAGCCT | Protein expression |
| HA-FGF11-R | TTTCTCGAGGGGGGCAGGGGGACTGGAAG | Protein expression |
| HA-IGF2-F | TTTAAGCTTCGCCACCATGGGAATCCCAATGGGGAAGT | Protein expression |
| HA-IGF2-R | CTCGAGCGGCCGCTTCCGATTGCTGGCCATCT | Protein expression |
| IGF1R-1-T | TCGAGTTGCTCCATTTGAGAGACACGCTGGCGACACACTCCGTCCATCCGACGC | Reporter construct |
| IGF1R-1-B | GGCCGCGTCGGATGGACGGAGTGTGTCGCCAGCGTGTCTCTCAAATGGAGCAAC | Reporter construct |
| IGF1R-1-MT | TCGAGTTGCTCCATTTGAGAGACACGCTGGCGACAGTGAGGGTCCATCCGACGC | Reporter construct |
| IGF1R-1-MB | GGCCGCGTCGGATGGACCCTCACTGTCGCCAGCGTGTCTCTCAAATGGAGCAAC | Reporter construct |
| IGF1R-2-T | TCGAGCCTTCAGGTCCACCCTCTCCCCTTTCTGCTCACTCCAAGAAACTTCTGC | Reporter construct |
| IGF1R-2-B | GGCCGCAGAAGTTTCTTGGAGTGAGCAGAAAGGGGAGAGGGTGGACCTGAAGGC | Reporter construct |
| IGF1R-2-MT | TCGAGCCTTCAGGTCCACCCTCTCCCCTTTCTGCTGTGAGGAAGAAACTTCTGC | Reporter construct |
| IGF1R-2-MB | GGCCGCAGAAGTTTCTTCCTCACAGCAGAAAGGGGAGAGGGTGGACCTGAAGGC | Reporter construct |
| MERTK-T | TCGAGACATCGACCCTGACTCTATAATTGCCTCCTGCACTCCCCGCGCTGCCGC | Reporter construct |
| MERTK-B | GGCCGCGGCAGCGCGGGGAGTGCAGGAGGCAATTATAGAGTCAGGGTCGATGTC | Reporter construct |
| MERTK-T-MU | TCGAGACATCGACCCTGACTCTATAATTGCCTCCTGGTGAGGCCGCGCTGCCGC | Reporter construct |
| MERTK-B-MU | GGCCGCGGCAGCGCGGCCTCACCAGGAGGCAATTATAGAGTCAGGGTCGATGTC | Reporter construct |
| MERTK-2-T | TCGAGAGTCCTGATGTGAGGAGAGGTGCGGGGAGACATTCCAAAAATCAAGCGC | Reporter construct |
| MERTK-2-B | GGCCGCGCTTGATTTTTGGAATGTCTCCCCGCACCTCTCCTCACATCAGGACTC | Reporter construct |
| MERTK-2-MT | TCGAGAGTCCTGATGTGAGGAGAGGTGCGGGGAGTGTAAGGAAAAATCAAGCGC | Reporter construct |
| MERTK-2-MB | GGCCGCGCTTGATTTTTCCTTACACTCCCCGCACCTCTCCTCACATCAGGACTC | Reporter construct |
| PKM2-T | TCGAGCCCACCCTCCACTCAGCTGTCCTGCAGCAAACACTCCACCCTCCACCGC | Reporter construct |
| PKM2-B | GGCCGCGGTGGAGGGTGGAGTGTTTGCTGCAGGACAGCTGAGTGGAGGGTGGGC | Reporter construct |
| PKM2-T-MU | TCGAGCCCACCCTCCACTCAGCTGTCCTGCAGCAAAGTGAGGACCCTCCACCGC | Reporter construct |
| PKM2-B-MU | GGCCGCGGTGGAGGGTCCTCACTTTGCTGCAGGACAGCTGAGTGGAGGGTGGGC | Reporter construct |
| FGFR1-T | TCGAGGAGGTTGCAGTGAGCCGAGATTGCGCCATTGCACTCCAGCCTGGGCAGC | Reporter construct |
| FGFR1-B | GGCCGCTGCCCAGGCTGGAGTGCAATGGCGCAATCTCGGCTCACTGCAACCTCC | Reporter construct |
| FGFR1-T-MU | TCGAGGAGGTTGCAGTGAGCCGAGATTGCGCCATTGGTGAGGAGCCTGGGCAGC | Reporter construct |
| FGFR1-B-MU | GGCCGCTGCCCAGGCTCCTCACCAATGGCGCAATCTCGGCTCACTGCAACCTCC | Reporter construct |
| FGFR1-2-T | TCGAGGGCTCCCCCGTACCTCATGGCTCAAACTACCACTCCTCAGTCGCTATGC | Reporter construct |
| FGFR1-2-B | GGCCGCATAGCGACTGAGGAGTGGTAGTTTGAGCCATGAGGTACGGGGGAGCCC | Reporter construct |
| FGFR1-2T-MU | TCGAGGGCTCCCCCGTACCTCATGGCTCAAACTACGTGAGGTCAGTCGCTATGC | Reporter construct |
| FGFR1-2B-MU | GGCCGCATAGCGACTGACCTCACGTAGTTTGAGCCATGAGGTACGGGGGAGCCC | Reporter construct |
| FGF11-T1 | TCGAGAGGTCAGGCCAAGAAGGGTGAAGGCCTCTTGCACTCCAGACCTCATAGC | Reporter construct |
| FGF11-B1 | GGCCGCTATGAGGTCTGGAGTGCAAGAGGCCTTCACCCTTCTTGGCCTGACCTC | Reporter construct |
| FGF11-T1-MU | TCGAGAGGTCAGGCCAAGAAGGGTGAAGGCCTCTTGGTGAGGAGACCTCATAGC | Reporter construct |
| FGF11-B1-MU | GGCCGCTATGAGGTCTCCTCACCAAGAGGCCTTCACCCTTCTTGGCCTGACCTC | Reporter construct |
| FGF11-T2 | TCGAGTCCTGGCACTGCTCCCAGGGGATCGGGTCTCCACTCCAGCTTTCTCAGC | Reporter construct |
| FGF11-B2 | GGCCGCTGAGAAAGCTGGAGTGGAGACCCGATCCCCTGGGAGCAGTGCCAGGAC | Reporter construct |
| FGF11-T2-MU | TCGAGTCCTGGCACTGCTCCCAGGGGATCGGGTCTCGTGAGGAGCTTTCTCAGC | Reporter construct |
| FGF11-B2-MU | GGCCGCTGAGAAAGCTCCTCACGAGACCCGATCCCCTGGGAGCAGTGCCAGGAC | Reporter construct |
| IGF2-T1 | TCGAGGAGAAACAATTGGCAAAATAAAGGAATTTGGCACTCCCCACCCCCCTGC | Reporter construct |
| IGF2-B1 | GGCCGCAGGGGGGTGGGGAGTGCCAAATTCCTTTATTTTGCCAATTGTTTCTCC | Reporter construct |
| IGF2-T1-M | TCGAGGAGAAACAATTGGCAAAATAAAGGAATTTGGGTGAGGCCACCCCCCTGC | Reporter construct |
| IGF2-B1-M | GGCCGCAGGGGGGTGGCCTCACCCAAATTCCTTTATTTTGCCAATTGTTTCTCC | Reporter construct |
| IGF2-T2 | TCGAGCCGAAAATAGCAACAACCCAGACTGGCTCCTCACTCCCTTTTCCATCGC | Reporter construct |
| IGF2-B2 | GGCCGCGATGGAAAAGGGAGTGAGGAGCCAGTCTGGGTTGTTGCTATTTTCGGC | Reporter construct |
| IGF2-T2-M | TCGAGCCGAAAATAGCAACAACCCAGACTGGCTCCTGTGAGGCTTTTCCATCGC | Reporter construct |
| IGF2-B2-M | GGCCGCGATGGAAAAGCCTCACAGGAGCCAGTCTGGGTTGTTGCTATTTTCGGC | Reporter construct |
| MAP3K3T | TCGAGATGAATTTGAAGACAAGCCAACAAACCCTGCACTCCAAAAAAGCAAAGC | Reporter construct |
| MAP3K3B | GGCCGCTTTGCTTTTTTGGAGTGCAGGGTTTGTTGGCTTGTCTTCAAATTCATC | Reporter construct |
| MAP3K3-MT | TCGAGATGAATTTGAAGACAAGCCAACAAACCCTGGTGAGGAAAAAAGCAAAGC | Reporter construct |
| MAP3K3-MB | GGCCGCTTTGCTTTTTTCCTCACCAGGGTTTGTTGGCTTGTCTTCAAATTCATC | Reporter construct |
| IL1RN-T-WI | TCGAGCACTGCTGCCCAGCCTCCAAGCTCCATCTCCACTCCAGATTTTTTACGC | Reporter construct |
| IL1RN-B-WI | GGCCGCGTAAAAAATCTGGAGTGGAGATGGAGCTTGGAGGCTGGGCAGCAGTGC | Reporter construct |
| IL1RN-T-MU | TCGAGCACTGCTGCCCAGCCTCCAAGCTCCATCTCGTGAGGAGATTTTTTACGC | Reporter construct |
| IL1RN-B-MU | GGCCGCGTAAAAAATCTCCTCACGAGATGGAGCTTGGAGGCTGGGCAGCAGTGC | Reporter construct |
| DSTYK-T1 | TCGAGGAGGTTGCAGTGAGCCGAGATCATGCCATTGCACTCCAGCCTGGGCGGC | Reporter construct |
| DSTYK-B1 | GGCCGCCGCCCAGGCTGGAGTGCAATGGCATGATCTCGGCTCACTGCAACCTCC | Reporter construct |
| DSTYK-T1-MU | TCGAGGAGGTTGCAGTGAGCCGAGATCATGCCATTGGTGAGGAGCCTGGGCGGC | Reporter construct |
| DSTYK-B1-MU | GGCCGCCGCCCAGGCTCCTCACCAATGGCATGATCTCGGCTCACTGCAACCTCC | Reporter construct |
| DSTYK-T2 | TCGAGAGGCTGCATTGAGCCAAGATCACACCACTGCACTCCAGCCTGGGCAGGC | Reporter construct |
| DSTYK-B2 | GGCCGCCTGCCCAGGCTGGAGTGCAGTGGTGTGATCTTGGCTCAATGCAGCCTC | Reporter construct |
| DSTYK-T2-MU | TCGAGAGGCTGCATTGAGCCAAGATCACACCACTGGTGAGGAGCCTGGGCAGGC | Reporter construct |
| DSTYK-B2-MU | GGCCGCCTGCCCAGGCTCCTCACCAGTGGTGTGATCTTGGCTCAATGCAGCCTC | Reporter construct |
| EPO-T | TCGAGTCTCAGCTCAGCGCCAGCCTGTCCCATGGACACTCCAGTGCCAGCAAGC | Reporter construct |
| EPO-B | GGCCGCTTGCTGGCACTGGAGTGTCCATGGGACAGGCTGGCGCTGAGCTGAGAC | Reporter construct |
| EPO-T-MU | TCGAGTCTCAGCTCAGCGCCAGCCTGTCCCATGGAGTGAGGAGTGCCAGCAAGC | Reporter construct |
| EPO-B-MU | GGCCGCTTGCTGGCACTCCTCACTCCATGGGACAGGCTGGCGCTGAGCTGAGAC | Reporter construct |
| OSMR-T | TCGAGAGGTTAAAGGCCAGAGGCTATGGAACTTAACACTCCCCATTGGAGCAGC | Reporter construct |
| OSMR-B | GGCCGCTGCTCCAATGGGGAGTGTTAAGTTCCATAGCCTCTGGCCTTTAACCTC | Reporter construct |
| OSMR-T-MU | TCGAGAGGTTAAAGGCCAGAGGCTATGGAACTTAAGTGAGGCCATTGGAGCAGC | Reporter construct |
| OSMR-B-MU | GGCCGCTGCTCCAATGGCCTCACTTAAGTTCCATAGCCTCTGGCCTTTAACCTC | Reporter construct |
| JAK3-T1 | TCGAGTGCCAAGGCCAGCGGCATCCTGCCTGTGTACCACTCCCTCTTTGCTCGC | Reporter construct |
| JAK3-B1 | GGCCGCGAGCAAAGAGGGAGTGGTACACAGGCAGGATGCCGCTGGCCTTGGCAC | Reporter construct |
| JAK3-T1-MU | TCGAGTGCCAAGGCCAGCGGCATCCTGCCTGTGTACGTGAGGCTCTTTGCTCGC | Reporter construct |
| JAK3-B1-MU | GGCCGCGAGCAAAGAGCCTCACGTACACAGGCAGGATGCCGCTGGCCTTGGCAC | Reporter construct |
| JAK3-T2 | TCGAGTGCCTTCACTGCTCACCCAGAGGGCAAACACCACTCCCTGTCCTTTTGC | Reporter construct |
| JAK3-B2 | GGCCGCAAAAGGACAGGGAGTGGTGTTTGCCCTCTGGGTGAGCAGTGAAGGCAC | Reporter construct |
| JAK3-T2-MU | TCGAGTGCCTTCACTGCTCACCCAGAGGGCAAACACGTGAGGCTGTCCTTTTGC | Reporter construct |
| JAK3-B2-MU | GGCCGCAAAAGGACAGCCTCACGTGTTTGCCCTCTGGGTGAGCAGTGAAGGCAC | Reporter construct |
| ABL2-T1 | TCGAGGCTTGCAGTGAGCCCAGATCGCGCCACTGCACTCCAGCCTGGGTGACGC | Reporter construct |
| ABL2-B1 | GGCCGCGTCACCCAGGCTGGAGTGCAGTGGCGCGATCTGGGCTCACTGCAAGCC | Reporter construct |
| ABL2-T1-MU | TCGAGGCTTGCAGTGAGCCCAGATCGCGCCACTGGTGAGGAGCCTGGGTGACGC | Reporter construct |
| ABL2-B1-MU | GGCCGCGTCACCCAGGCTCCTCACCAGTGGCGCGATCTGGGCTCACTGCAAGCC | Reporter construct |
| ABL2-T2 | TCGAGAGGTTGCAGTGAGCTGAGATCGCGCCGCTGCACTCCAGCCTAAGTGAGC | Reporter construct |
| ABL2-B2 | GGCCGCTCACTTAGGCTGGAGTGCAGCGGCGCGATCTCAGCTCACTGCAACCTC | Reporter construct |
| ABL2-T2-MU | TCGAGAGGTTGCAGTGAGCTGAGATCGCGCCGCTGGTGAGGAGCCTAAGTGAGC | Reporter construct |
| ABL2-B2-MU | GGCCGCTCACTTAGGCTCCTCACCAGCGGCGCGATCTCAGCTCACTGCAACCTC | Reporter construct |
| EFNA1-T | TCGAGAAAGAGGGACAGGCTGAAGAGAGGGACAGGCACTCCAAACCTGTCTTGC | Reporter construct |
| EFNA1-B | GGCCGCAAGACAGGTTTGGAGTGCCTGTCCCTCTCTTCAGCCTGTCCCTCTTTC | Reporter construct |
| EFNA1-T-MU | TCGAGAAAGAGGGACAGGCTGAAGAGAGGGACAGGGTGAGGAAACCTGTCTTGC | Reporter construct |
| EFNA1-B-MU | GGCCGCAAGACAGGTTTCCTCACCCTGTCCCTCTCTTCAGCCTGTCCCTCTTTC | Reporter construct |
| IL1R1-T-WI | TCGAGCTTGATTTCAGGTCAATAACGGTCCCCCCTCACTCCACACTGGCACGGC | Reporter construct |
| IL1R1-B-WI | GGCCGCCGTGCCAGTGTGGAGTGAGGGGGGACCGTTATTGACCTGAAATCAAGC | Reporter construct |
| IL1R1-T-MU | TCGAGCTTGATTTCAGGTCAATAACGGTCCCCCCTGTGAGGACACTGGCACGGC | Reporter construct |
| IL1R1-B-MU | GGCCGCCGTGCCAGTGTCCTCACAGGGGGGACCGTTATTGACCTGAAATCAAGC | Reporter construct |
